# Hepatic Steatosis in Patients with Celiac Disease: The Role of Packaged Gluten-Free Foods

**DOI:** 10.3390/nu14142942

**Published:** 2022-07-18

**Authors:** Alberto Raiteri, Alessandro Granito, Chiara Faggiano, Alice Giamperoli, Teresa Catenaro, Giulia Negrini, Francesco Tovoli

**Affiliations:** 1 Division of Internal Medicine, Hepatobiliary and Immunoallergic Diseases, IRCCS Azienda Ospedaliero, University of Bologna, 40138 Bologna, Italy; alberto.raiteri@studio.unibo.it (A.R.); alessandro.granito@unibo.it (A.G.); chiara.faggiano@aosp.bo.it (C.F.); 2 Department of Medical and Surgical Sciences, University of Bologna, 40138 Bologna, Italy; alice.giamperoli@studio.unibo.it (A.G.); teresa.catenaro@studio.unibo.it (T.C.); giulia.negrini2@studio.unibo.it (G.N.)

**Keywords:** celiac disease, non-alcoholic fatty liver disease, steatosis, gluten-free diet, gluten

## Abstract

Background: An increased risk of nonalcoholic fatty liver disease (NAFLD) in patients with celiac disease (CD) adhering to a gluten-free diet (GFD) was recently reported. The nutritional composition of packaged gluten-free foods (PGFF) has been proposed as a possible cause. This hypothesis has not been investigated further, since a systematic structural nutritional interview for all patients would be problematic in clinical practice. Methods: We administered a simple questionnaire based on a Recency, Frequency, and Monetary value (RFM) analysis (a cornerstone of direct marketing segmentation) to consecutive CD patients on a GFD for >6 months and verified its association with NAFLD. Subgroup analyses were performed to understand whether specific patterns of PGFF consumption were significantly associated with NAFLD. Results: Amongst 147 patients (female 82%, median age 42 years), 45 (30.6%) had NAFLD. Total RFM score (adjusted odds ratio = 1.223, 95% CI: 1.059–1.413, *p* = 0.006), body mass index, and total cholesterol and triglycerides were independently related to NAFLD, and “Bread and bakery” (*p* = 0.002), “salty convenience” (*p* = 0.005), and “sweet convenience” (*p* = 0.049) products were significantly related with NAFLD. Also, questions about the number of purchased PGFF in the last month (monetary value) and different categories of PGFF consumed in the last week (recency) were particularly able to identify NAFLD patients. Conclusions: The specific GFD dietary habits of CD patients were correlated with the degree of risk of NAFLD. Information was obtained through a questionnaire which could be used in clinical practice to favor a patient-tailored approach and in future studies to verify the reproducibility of our results in different geographical areas.

## 1. Introduction

Celiac disease (CD) is an immune-mediated disease requiring a lifelong gluten-free diet (GFD) [1]. While GFD is a safe therapeutic option, it can lead to adverse metabolic alterations [2,3] and increase the cardiovascular risk [4]. An increased risk of nonalcoholic fatty liver disease (NAFLD) in both children and adults with CD on a GFD, compared to the general population, emerged in the last years [5,6]. 

NAFLD has been related to an increased risk of liver cirrhosis and primary liver cancer [7]. Alarmingly, recent evidence suggested that NAFLD also increases long-term mortality for cancer, liver disease, and cardiovascular disease in children and young adults [8]. Since most CD patients start on a GFD at a young age, pathogenic studies are needed to understand this phenomenon and reduce the risk of liver and all-cause related morbidity and mortality. 

The pathogenic link between GFD and NAFLD, however, has yet to be fully elucidated. Persistent gut–liver axis alterations and an unfavorable composition of the GFD have been proposed as potential etiological factors [9]. 

While reliable and non-invasive assessments of altered intestinal permeability can be difficult in daily clinical practice, the intake of packaged gluten-free foods (PGFF) can be analyzed. 

Until now, the nutritional profile of CD patients adhering to a GFD has been assessed by means of detailed nutritional interviews. While this approach is particularly indicated in clinical studies, it can be time consuming and difficult to apply in real-life clinical practice, in which physicians are required to examine a large number of patients in a relatively short period. 

Simple tools are consequently needed to help clinicians in the identification of CD patients at high risk for NAFLD and thus needing a tailored nutritional intervention. 

Recency, Frequency, and Monetary value (RFM) analysis is the cornerstone of direct marketing segmentation. It is often used in marketing studies to identify patterns of customer behavior and help in targeting the most sensitive audience for a specific product.

The three variables of this model represent three different dimensions of customer behavior: Recency (R) is the time since the last purchase and mostly represents the customer’s fidelity to a product; Frequency (F) is the number of purchases in a period of time, irrespective of the monetary value of single purchase; and Monetary value (M) is the total amount of money spent in a period of time, irrespective of the number of single purchases. 

In RFM analysis, the most common scoring method aims to sort customers in descending order (best to worst). To this end, the variables R, F, and M are split into quintiles. 

The primary aim of our study was to verify whether dietary habits, measured according to an RFM analysis, were independently associated with NAFLD in CD patients adhering to a GFD. The secondary aim was to verify whether specific categories of PGFF were associated with a different risk of NAFLD. 

## 2. Materials and Methods

### 2.1. Study Design

Prospective interventional clinical study enrolling CD patients following a GFD, to be evaluated for NAFLD and PGFF consumption (according to a dedicated RFM analysis).

Inclusion criteria:(1) CD diagnosed according to the criteria of the North American Society for Pediatric Gastroenterology, Hepatology, and Nutrition [10]; (2) GFD started at least 6 months before enrolment; (3) biochemical laboratory tests performed less than 2 months before enrolment.

Exclusion criteria:(1) incomplete compliance to the GFD; (2) ongoing or suspected pregnancy; (3) daily alcohol consumption of more than two alcoholic units for women and four units for men; (4) use of potentially steatogenic drugs, including anastrozole, corticosteroids, and cytotoxic agents; (5) any other known chronic liver disease at the time of enrolment (e.g., viral. autoimmune, metabolic, and storage diseases).

Compliance to the GFD was recorded as satisfactory if all the following criteria were respected: (1) absence of reported intentional or accidental gluten ingestion in the last 6 months [11]; (2) no CD-related symptoms [12]; (3) negative anti-transglutaminase IgA antibodies [10]; and (4) Biagi score < 3 points [13].

### 2.2. Subclassification of PGFF

Gluten-free products were classified according to their distinct nutritional qualities and costs, as proposed by Missbach et al. [14]. Briefly, these categories included: flour/bake mix (G1), bread and bakery products (G2), pasta and cereal-based food (G3), cereals (breakfast) (G4), cookies and cakes (breakfast) (G5), snacks (G6), and convenience foods (G7). Based on papers with a methodology similar to that of Missbach et al. [14] but reporting heterogeneous compositions in snacks and convenience foods [15,16,17,18], the last two groups were subdivided into sweet snacks (G6a), salty snacks (G6b), salty convenience (G7a) and sweet convenience (G7b) to ensure a better corroboration with existing evidence.

### 2.3. RFM Analysis

The three elements of Recency, Frequency, and Monetary value were adapted to this context, maintaining the original concept. For each category of PGFF, the following information was recorded: monetary value (defined as the number of packages bought in a month), frequency (defined as the average number of days in a week in which a given product category was consumed), and recency (defined as having consumed or not this product in the last week). A copy of the questionnaire administered to the patients (translated into English) is provided in the Supplementary Materials.

Total M, F, and R scores were obtained by summing the partial M, F, and R values derived from the groups. As previous explained, these total scores were subsequently divided into quintiles, so that each patient received a grade 1 to 5 for each variable and a total score of 3 to 15 as the sum of the three variables, according to a well-established methodology.

### 2.4. Ultrasound Evaluation and Diagnosis of NAFLD

Patients underwent an abdominal ultrasound examination during a scheduled visit to our CD outpatient clinic. For all patients, ultrasound evidence of liver steatosis was investigated by a member of our team with a minimum of 5 years’ experience in ultrasound imaging and who performs at least 350 ultrasound examinations per year. Ultrasound evidence of liver steatosis was defined according to the Hamaguchi criteria [19]. Assessment of steatosis was performed with real-time imaging. The ultrasound operator was blinded to the dietary information.

Nonalcoholic fatty liver disease was diagnosed according to the guidelines of the European Association for the Study of the Liver [7]. Presence of hepatic steatosis and exclusion of differential diagnoses were both required. Systematic histological confirmation of NAFLD by means of liver biopsies outside of clinical trials (as was the case for our study) is not supported by the current guidelines and would have posed ethical concerns [7,20]. Instead, liver biopsies were reserved for patients with noninvasive markers (such as NAFLD fibrosis score and Fibrosis4-score) suggestive of advanced liver disease [7,20].

### 2.5. Sample size Calculation

Previous descriptions in the literature reported a prevalence of NAFLD in CD patients of about 34% [6]. No RFM analyses have been previously reported in CD patients, so the expected RFM values were estimates. Based on the nutritional intake of our geographical area, a mean RFM value of 10 could be expected in our study population. Hypothesizing a mean RFM of 11 in NAFLD patients and 9 in patients without NAFLD, with a type I error α level of 0.05, the inclusion of a minimum 141 patients produced a power >90%.

### 2.6. Ethics

This study was approved by our Institutional Review Board (protocol 192/2017/O/Sper) and performed according to the Declaration of Helsinki guidelines. All patients signed an informed consent before enrolling into the study.

### 2.7. Statistical Analysis

Continuous variables are expressed as medians and interquartile ranges. Categorical variables are expressed as frequencies.

Group comparisons were performed with the Mann–Whitney test. Categorical variables were evaluated using the two-tailed Fisher’s test.

Binary logistic regression was performed using the presence of NAFLD as the independent variable. Variables for which the association with NAFLD in the univariate analysis was *p* < 0.10 were entered into the multivariate models to identify factors independently associated with NAFLD. A sensitivity analysis was performed excluding patients which were already overweight or obese at the time of the diagnosis of CD, to minimize the possible impact of undetected hepatic steatosis prior to the beginning of GFD. A *p* < 0.05 was considered to be the cut-off for statistical significance.

The statistical analysis was performed with SPSS version 23.0 (SPSS Inc., Chicago, IL, USA).

## 3. Results

### 3.1. Study Population

**Figure 1:**
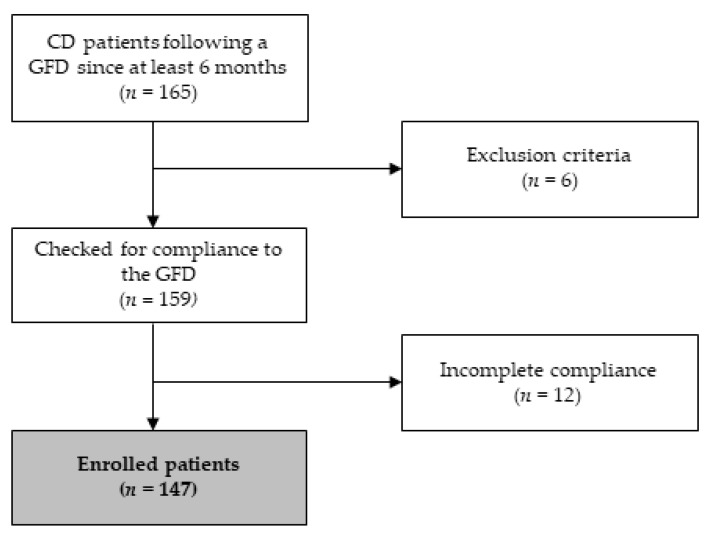
Patient flow chart. CD: celiac disease; GFD: gluten-free diet.

One hundred and sixty-five CD patients were evaluated; after applying the inclusion and exclusion criteria, a total of 147 patients were eligible for this study (Figure 1).

Female gender was predominant, and the median age at enrolment was 42 years (interquartile range 28–56; Table 1). The majority of patients had been on a GFD for more than 5 years (113 cases, 76.9%), 22 (15.0%) for between 2–5 years, and 12 (8.2%) for less than 2 years. Forty cases (27.2%) had been diagnosed in childhood (<15 years). Only 9 (6.1%) patients were overweight or obese at diagnosis.

### 3.2. Consumption of Packaged Gluten-Free Foods

All patients acquired at least one PGFF in the week preceding the enrollment. The groups of PGFF with a more prevalent recency were pasta and cereal-based food (G3-93.2%), sweet snacks (G6a-72.8%), cookies and cakes (G5-68.7%), and bread and bakery products (G2-68.0%). Inb contrast, the groups with a recency <50% were: sweet convenience (G7b-25.9%), cereals for breakfast (G4-26.5%), salty convenience (G7a-36.7%), and sweet snacks (G6a-42.2%). The highest partial F scores were recorded for pasta and cereal-based food (G3-median 4, IQR 3–7), cookies and cakes (G5-median 3, IQR 1–6), and salty snacks (G6b-median 3, IQR 1–5). The highest partial M scores were in the following subgroups: pasta and cereal-based food (G3-median 4, IQR 3–7), bread and bakery products (G2-median 3, IQR 1–5), cookies and cakes (G5-median 2, IQR 1–4), and salty snacks (G6b-median 2, IQR 1–3).

The median total raw R, F, and M values were 5 (IQR 4–6), 21 (IQR 16–26), and 20 (14–26), respectively. Quintile thresholds are described in Table 2.

**Figure 2:**
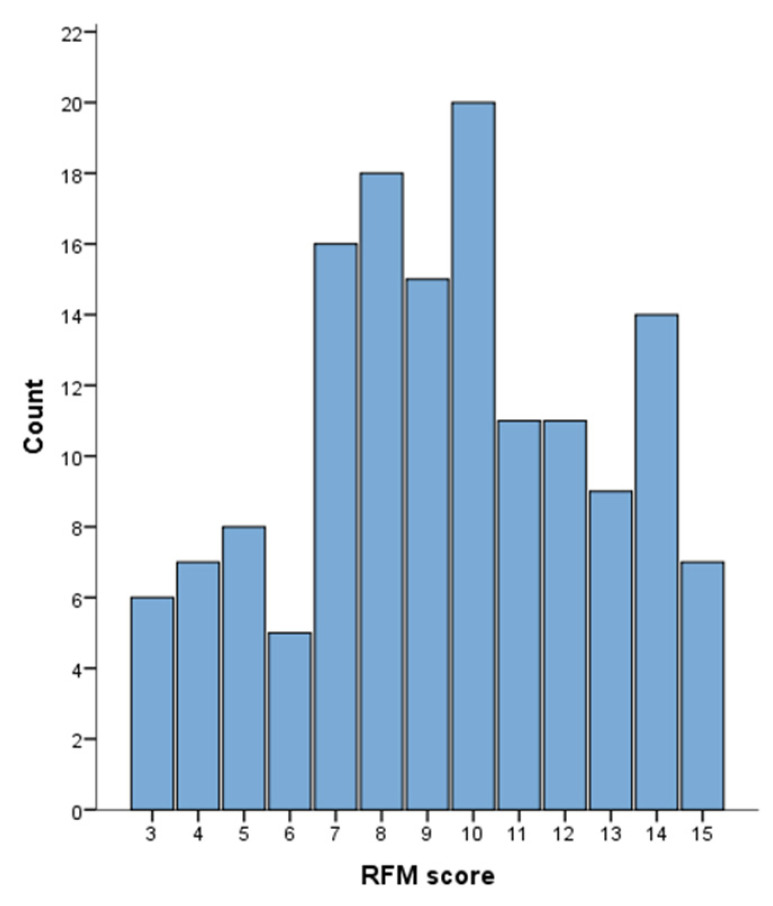
Distribution of the Recency, Frequency, Monetary value (RFM) score in the whole study population (*n* = 147).

Following the standardization in quintiles and summing the standardized R, F, and M scores, the median RFM score was 9 (IQR 7–12), with 6 (4.1%) patients at the minimum score (RFM = 3) and 7 (4.8%) patients reaching the highest score (RFM = 15). Figure 2 presents the distribution of the RFM score in the study population.

### 3.3. Prevalence and Relative Risk of NAFLD

NAFLD was found in 45 (30.6%) enrolled patients. The presence of NAFLD was associated with a significantly higher RFM score (10.7 ± 2.9 vs. 8.8 ± 3.2, *p* = 0.001).

Binary logistic regression confirmed RFM score as an independent risk factor for NAFLD (adjusted odds ratio = 1.223, 95% CI: 1.059–1.413, *p* = 0.006). Other factors independently related to NAFLD were body mass index and total cholesterol and triglycerides (Table 3). 

Additional models were created using separate R, F, and M scores. Both R (odds ratio 1.699, 95% CI: 1.155–2.498, *p* = 0.007) and M (odds ratio 1.549, 95% CI: 1.116–2.151, *p* = 0.009), but not F (odds ratio 1.143, 95% CI: 0.853–1.531, *p* = 0.370), were confirmed as independent predictors of NAFLD. After the sensitivity analysis, the RFM score remained associated with NAFLD (*p* = 0.010).

### 3.4. Role of Single PGFF Categories

**Figure 3:**
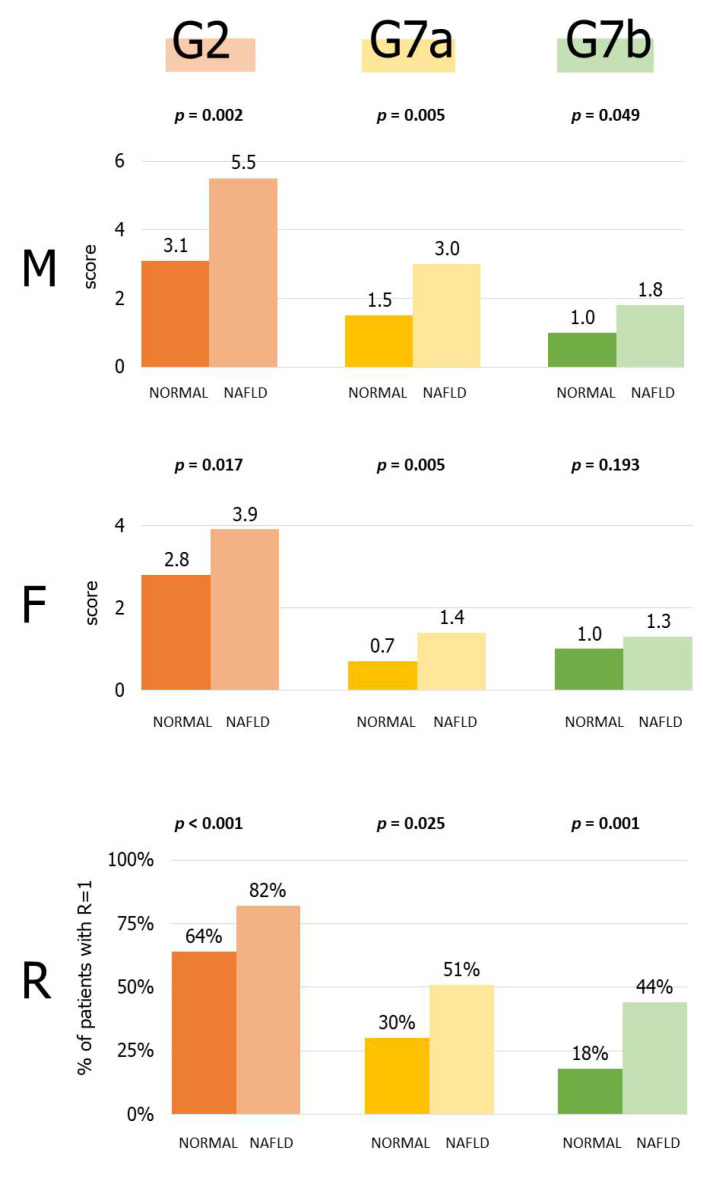
Recency (R), frequency (F), and monetary value (M) scores of bread/bakery (G2), salty (G7a), and sweet convenience products (G7b) in patients with and without nonalcoholic fatty liver disease (the latter category defined as “normal”).

Since the RFM score was significantly correlated with steatosis, we performed additional analyses to verify whether steatosis was associated with particular PGFF groups. NAFLD patients had significantly higher partial R, F, and M scores for the bread and bakery products (G2) and salty convenience (G7a) categories. For the sweet convenience category (G7b), R and M (but not F) scores were significantly higher in the NAFLD group (Figure 3). No associations were found for the remaining groups.

## 4. Discussion

NAFLD in CD patients adhering to a GFD was recently described, but its determinants remain unclear. NAFLD was also reported in a sizeable portion of lean subjects and independently from typical metabolic risk factors [6].

We demonstrated that: (1) a relative increasing in PGFF consumption was significantly related with NAFLD; and (2) not all PGFF were associated equally with NAFLD; rather, specific patterns of consumption were particularly at risk.

In general, PGFF are known to have unfavorable nutritional characteristics compared to their gluten-containing counterparts, particularly in terms of their lipid [21,22,23] and carbohydrate [21,24] contents. Whether PGFF subclasses are “less healthy” remains a matter of debate. In their original investigation of Austrian PGFF, Missbach et al. [14] did not find significant nutritional differences in bread and snacks. However, other studies conducted with similar methodologies showed that Canadian [15,25], Norwegian [16], Slovenian [17], Moroccan [18], and Brazilian [26] gluten-free breads had higher carbohydrate and saturated fat contents compared to gluten-containing breads. Similar differences were also reported for sweet convenience foods, another category which was particularly associated with NAFLD in our study. In contrast, nutritional differences in other categories were less relevant.

Until now, no study has verified whether these nutritional differences also translate into actual metabolic alterations. Thus, we provided novel information which could prove useful to improve primary and secondary prevention of NAFLD in CD patients.

Our results substantially confirmed the hypothesis which attributes hepatic primarily to the PGFF composition. Two different mechanisms may contribute to the steatogenesis. First, increased amounts of carbohydrate and fats in the small bowel can lead to a larger quantity of these nutrients arriving at the liver through portal flow. Second, short-chain fatty acids might interact with the gut microbiota, leading to dysregulated production of acetate and propionate (two regulators of de novo lypogenesis in the liver) [27].

Our results also provide insights which might be useful in the nutritional management of CD patients, especially for those at risk of steatosis. Three patterns of nutritional behaviors should be well monitored. The first is the purchase/consumption of large amounts of PGFF per month (Monetary value). The second is the purchase/consumption of a great variety of different PGFF during the last week (Recency), meaning a fondness for such products, and finally, a fondness for specific PGFFs which are particularly related to NAFLD (in our study: bread and bakery products, and conveniences—both sweet and salty). This information is relevant to provide nutritional tips and suggestions of new food approaches for CD patients to avoid metabolic disorders, including NAFLD. For instance, patients should be encouraged to increase their consumption of olive oil, legumes, unrefined cereals, fruits, and vegetable and introduce pseudocereals as a source of complex carbohydrates, protein, fiber, fatty acids, vitamins, and minerals [28].

Our study has some limitations deserving discussion. First, RFM analyses cannot provide detailed nutritional information; rather, they can be used as a screening tool to identify patients who should receive a dedicated and comprehensive nutritional assessment, paired with tailored alimentary prescriptions. Second, our findings need external validation (possibly from different geographical areas), as the nutritional composition of the PGFF might vary internationally according to customer preferences. Third, our population did not include patients with NAFLD or severe liver fibrosis, so it is impossible to verify if specific patterns of PGFF consumption are associated with a more aggressive phenotype of NAFLD. The lack of such patients, however, was largely expected due to the relatively young age of our cohort. Still, the slowly evolving nature of NAFLD does not rule out that a small proportion of patients with untreated NAFLD will eventually transition to severe forms of liver disease over time and incur into the risk of liver-related death [7].

In conclusion, we found that the GFD dietary behavior of CD patients correlated with NAFLD. Information was obtained through a simple, reproducible, and time-saving questionnaire which can be used in clinical practice to create patient-tailored approaches and in future studies to validate our results in different geographical areas.

## Figures and Tables

**Table 1:** Characteristics of the study population. Continuous variables are expressed as median (interquartile range), categorical variables as frequencies (%).

Variable	
Age (years)	42 (28–56)
Female sex	107 (81.7)
Body mass index (BMI)	22.0 (19.8–24.0)
BMI categorization	
-Underweight (<18.5)	9 (6.9)
-Normal weight (18.5–25)	93 (71.0)
-Overweight (25–30)	24 (18.3)
-Obesity (>30)	5 (3.8)
Total cholesterol (mg/dL)	196 (171–220)
Total cholesterol >200 mg/dL	62 (47.3)
HDL cholesterol (mg/dL)	57 (53–64)
HDL cholesterol low ^1^	19 (14.5)
Triglycerides (mg/dL)	79 (64–98)
Triglycerides >150 mg/dL	8 (6.1)
Diabetes	11 (8.4)
AST (UI/L)	19 (16–24)
AST high ^2^	3 (2.0)
ALT (UI/L)	17 (13–22)
ALT high ^2^	10 (6.8)

HDL: high-density lipoproteins; AST: aspartate aminotransferase; ALT: alanine aminotransferase; ^1^ <50 mg/dL in women; <40 mg/dL in men. ^2^ >35 U/L in women; >50 U/L in men.

**Table 2:** Threshold of the quintiles of the total R, F, and M scores. The total M, F, and R scores were obtained by summing the partial values from the single PGFF categories.

Raw scores	Q1	Q2	Q3	Q4	Q5
M	2–13	14–18	19–21	22–31	32–71
F	1–15	16–19	20–23	24–27	28–52
R	1–2	3	4	5	6–9

**Table 3:** Univariate and multivariate binary logistic regression to nonalcoholic fatty liver disease.

Univariate Analysis		Variable	Multivariable Analysis	
Exp(B) (95% CI)	*p*		Exp(B) (95% CI)	*p*
1.050 (1.023–1.078)	<0.001	Age (years)	1.039 (1.007–1.072)	0.017
0.399 (0.160–0.995)	0.049	Sex (F = 1)	0.744 (0.204–2.714)	0.654
1.294 (1.137–1.473)	<0.001	Body mass index	1.228 (1.053–1.432)	0.009
1.013 (1.002–1.024)	0.023	Total cholesterol (mg/dL)	1.012 (0.998–1.027)	0.099
0.963 (0.932–0.996)	0.028	HDL cholesterol (mg/dL)	0.996 (0.986–1.005)	0.375
1.034 (1.016–1.051)	<0.001	Triglycerides (mg(dL)	1.030 (1.010–1.050)	0.003
2.197 (0.628–7.688)	0.218	Diabetes (No = 0, Yes = 1)	-	-
1.080 (1.019–1.144)	0.009	AST (UI/L) *	-	-
1.046 (1.010–1.085)	0.013	ALT (UI/L)	1.041 (0.970–1.017)	0.266
1.166 (1.030–1.321)	0.016	RFM score (units)	1.242 (1.057–1.459)	0.008

CI, confidence interval; HDL, high-density lipoproteins; AST, aspartate aminotransferase; ALT, alanine aminotransferase. * Not included in the multivariable analysis for colinearity.

## Data Availability

The data presented in this study are available on request from the corresponding author. The data are not publicly available due to privacy restrictions.

## Supplementary Materials

The following supporting information can be downloaded at: https://www.mdpi.com/article/10.3390/nu14142942/s1.
