# Supplementary material for: Hepatic Steatosis in Patients with Celiac Disease: The Role of Packaged Gluten-Free Foods
Source: Nutrients. 2022 Jul 18;14(14):2942. doi: 10.3390/nu14142942 (PMC9316041; doi:10.3390/nu14142942)
Supplement: Supplementary file 1 [file nutrients-14-02942-s001.zip › nutrients-1819010-supplementary.pdf]

Gluten-free packaged product	How many packages of these products do you buy in 1 months? (Monetary value)	How many days per week (1 to 7) do you eat these products on at least one occasion?(Frequency)	Have you eaten at least one of these products in the last week? YES=1 NO=0 (Recency)
**G1. Flour/Bake mix**			
Flour, bake mix for cakes, bake mix for pizza, breadcrumbs			
**G2. Bread and bakery**			
Rustic bread, whole-grain bread, toast, buns, ciabatta bread, raisin bread, Scone, baguette, lye brezel, rusk, Crispbread, Wraps			
**G3. Pasta and cereal-based**			
Fusilli, Spaghetti, Penne, Lasagne sheets, Vermicelli, Tagliatelli, Cous Cous			
**G4. Cereals**			
Granola (chocolate), Granola (nuts), Cornflakes			
**G5. Cookies and Cakes (breakfast)**			
Biscuits, Cookie (chocolate), Cookie (whole-grain), Cookie (orange)			
**G6a. Snacks (sweet) and Cakes**			
Shortbread, Neapolitan wafers, Shortbread, Granola bar, Apple strudel, Muffin, Plumcake			
**G6b. Snacks (salty)**			
Cracker, Brezels, Grissini, Saltsticks, Wafers (plain)			
**G7a. Convenience (salty)**			
Pizza (salami), Pizza (margherita), Lasagne, Chicken Nuggets, Fish sticks, Soup (potato and leek), Soup (mushrooms)			
**G7b. Convenience (sweet)**			
Wafer-cone (icecream filling), Pudding (semolina), Baked pastry case, Wafer (Oblate), Rice Drink (natural), Flaky pastry, Frozen Cake (almond, chocolate)			
**TOTAL SCORE**			
